# Supplementary material for: “I don’t know if it makes a difference to safety?” perception vs actuality: A mixed-methods study on older adults’ experiences of home stair falls revealed during COVID-19 lockdown
Source: PLoS One. 2025 Jun 26;20(6):e0326850. doi: 10.1371/journal.pone.0326850 (PMC12200730; doi:10.1371/journal.pone.0326850)
Supplement: S1 Appendix — (DOCX) [file pone.0326850.s001.docx]

**S1 Appendix. Semi-structured topic guide used in interviews.**

Q1. Can you tell me a bit about your home?

Q2. Reflecting on a typical week before the pandemic. What were your reasons to leave the house?

Q2a. How did that change during home-confinement/lockdown?

Q3. Can you talk me through how you spent a typical day in lockdown?

Q3a. Do you feel you used your home stairs more during the lockdowns?

Q4. How did you feel during lockdown/home-confinement, when living on your own/when living with others?

Q5. In the survey you said you felt…ADD PHYSICAL ACIVITY LEVEL (“more physically active during lockdown”), why do you think this?

Q6. How do you feel about COVID-19 now when leaving the house?

Q7. Thinking about home stair falls. Have you experienced a new stair fall in your home since you completed the survey?

*Q7a(i). If you experienced a home stair fall,* do you feel you fell on your stairs more during lockdown?

*Q7a(ii). If you experienced a near home stair fall,* do you feel you nearly fell on your stairs more during lockdown?

*Q7a (iii). If you haven’t experienced a home stair fall,* why do you think you haven’t experienced a stair fall at home?

Q8. Reflecting about the home stair fall/near home stair fall that’s most memorable, could you talk me through what happened?

Q8a. What impact has this had on you?

Q9. Thinking about your home stairs, you said that they were…ADD SURVEY RESPONSE (“safe” or “unsafe”). What key features make your stairs safe? Or What key features make your stairs a hazard?

Q9a. What barriers do you have to making your stairs safe?

Q10. Is there anything else you feel is important to mention which we have not covered yet?
