# Supplementary material for: “I don’t know if it makes a difference to safety?” perception vs actuality: A mixed-methods study on older adults’ experiences of home stair falls revealed during COVID-19 lockdown
Source: PLoS One. 2025 Jun 26;20(6):e0326850. doi: 10.1371/journal.pone.0326850 (PMC12200730; doi:10.1371/journal.pone.0326850)
Supplement: S2 Appendix — (DOCX) [file pone.0326850.s002.docx]

**S2 Appendix. Stair assessment protocol for evaluating staircase safety:**

**Visual Assessment**

**Location of dwelling**

Participant details

House number

Postcode

**Position at start of fall**

At the top

Near the top

Near the middle

Near the bottom

At the bottom

Don't know

**Stair type**

Straight stairs

Alternating tread stairs

Turning stairs (quarter landing or turn through 90°/180° to a second flight)

Narrow steps on one side (winders)

Spiral/helical stairs

Landing between floor levels

Don't know

Other

**Number of steps**

**Visibility through gaps**

Yes

No

Don't know

**Type of banister/handrails (Right side)**

Wall, no handrail

Wall with handrail

Banister with vertical rails

Ranch-style banister

Banister with intricate patterning

Solid banister

No banister/handrail/wall

Don't know

Other (add description)

**Type of banister/handrails (Left side)**

Wall, no handrail

Wall with handrail

Banister with vertical rails

Ranch-style banister

Banister with intricate patterning

Solid banister

No banister/handrail/wall

Don't know

Other (add description)

**Stair appearance**

Plain carpet

Pattern carpet

Exposed wood

Exposed metal or concrete

Lino/vinyl

Don't know

Other

**Damage or wear**

Broken or missing tread

Broken guarding

Broken handrail

Loose handrail

Damaged stair covering

Items left on steps

**Stair characteristics (Likert scale)**

Too steep

Too narrow

Poorly lit

Needs repair

Handrail needs repair

Covering needs repair

Stairs are safe to use

Visibility

**Artificial lighting**

Two-way switch available at both top and bottom

Steps well-lit with no shadows hiding nosing

Easy to change bulbs

Bulbs covered with diffused shade

**Daylight/windows over stairs**

Steps well-lit with no shadows hiding nosing

Windows reachable for cleaning

Windows operable (open/close)

Curtains/hangings manageable

No glare from windows when using stairs

Surrounding Environment

**Room type at the top of stairs**

Landing

Hall/lobby

Bedroom

Loft/attic

Living/dining area

Kitchen/utility room

Bathroom/toilet

Other

**Moveable item at top of stairs**

Yes

No

**Room type at bottom of stairs**

Landing

Hall/lobby

Bedroom

Loft/attic

Living/dining area

Kitchen/utility room

Bathroom/toilet

Other

**Moveable item at bottom of stairs**

Yes

No

**Measurements and Dimensions**

**Participant's height**

**Participant's shoe size**

**Measure risings/goings**

**Handrail height (mm)**

**Handrail cross-section design**

1½” Round

2½” Round

3” Round

2½” Decorative

3” Decorative

2½” Tapered

3” Tapered

**Stair width (mm)**

**Foot overhang (mm)**

**Accident Assessment**

**Past incidents**

**Has anyone else fallen on these stairs?**

Yes

No

Don't know

**Relation to the accident participant**

Myself

Husband/wife/partner

Son/daughter

Friend/visitor/stranger

Other

**Usual stair negotiation (Up)**

Use handrail

Sideways placement of body

Glance at feet

Step over step

Step by step

Other

**Usual stair negotiation (Down)**

Use handrail

Sideways placement of body

Glance at feet

Step over step

Step by step

Other
